# Impact of the Urban Reproductive Health Initiative on family planning uptake at facilities in Kenya, Nigeria, and Senegal

**DOI:** 10.1186/s12905-017-0504-x

**Published:** 2018-01-05

**Authors:** Jennifer Winston, Lisa M. Calhoun, Meghan Corroon, David Guilkey, Ilene Speizer

**Affiliations:** 1 Carolina Population Center, University of North Carolina at Chapel Hill, Campus Box 8120, Chapel Hill, NC 27599 USA; 2 Department of Economics and Carolina Population Center, University of North Carolina at Chapel Hill, Chapel Hill, NC USA; 3 Department of Maternal and Child Health, Gillings School of Global Public Health, and Carolina Population Center, University of North Carolina at Chapel Hill, Chapel Hill, NC USA

**Keywords:** Family planning, Facility improvements, Nigeria, Kenya, Senegal, Urban

## Abstract

**Background:**

The 2012 London Summit on Family Planning set ambitious goals to enable 120 million more women and adolescent girls to use modern contraceptives by 2020. The Urban Reproductive Health Initiative (URHI) was a Bill & Melinda Gates Foundation funded program designed to help contribute to these goals in urban areas in India, Kenya, Nigeria, and Senegal. URHI implemented a range of country-specific demand and supply side interventions, with supply interventions generally focused on improved service quality, provider training, outreach to patients, and commodity stock management. This study uses data collected by the Measurement, Learning & Evaluation (MLE) Project to examine the effectiveness of these supply-side interventions by considering URHI’s influence on the number of family planning clients at health facilities over a four-year period in Kenya, Nigeria, and Senegal.

**Methods:**

The analysis used facility audits and provider surveys. Principal-components analysis was used to create country-specific program exposure variables for health facilities. Fixed-effects regression was used to determine whether family planning uptake increased at facilities with higher exposure. Outcomes of interest were the number of new family planning acceptors and the total number of family planning clients per reproductive health care provider in the last year.

**Results:**

Higher program component scores were associated with an increase in new family planning acceptors per provider in Kenya (β = 18, 95% CI = 7–29), Nigeria (β = 14, 95% CI = 8–20), and Senegal (β = 7, 95% CI = 3–12). Higher scores were also associated with more family planning clients per provider in Kenya (β = 31, 95% CI = 7–56) and Nigeria (β = 26, 95% CI = 15–38), but not in Senegal.

**Conclusions:**

Supply-side interventions have increased the number of new family planning acceptors at facilities in urban Nigeria, Kenya, and Senegal and the overall number of clients in urban Nigeria and Kenya. While tailoring to the local environment, programs seeking to increase family planning use should include components to improve availability and quality of family planning services, which are part of a rights-based approach to family planning programming.

## Background

The 2012 London Summit on Family Planning, now known as the FP2020 Initiative, called for a range of global commitments to enable 120 million more women and adolescent girls to use modern contraceptives by 2020 [1]. Central to this goal is FP2020’s recognition of “the fundamental right of individuals to decide, freely and for themselves, whether, when, and how many children to have” [2]. Much of the programming prior to the London Summit focused on specific quality interventions including: ensuring availability of a range of methods, training of providers, inclusion of follow-up/continuity mechanisms for FP, and integration of FP with maternal, newborn, and child health services [3]. Recently, Jain has proposed that the typical quality of care strategies be expanded with a rights-based lens to promote interventions that are more responsive to the needs of women and girls as well as to encourage measurement that is more balanced to what rights-based family planning programs are undertaking [4]. This rights-based approach to family planning seeks to address women and girls’ ability (agency, autonomy and empowerment) to obtain contraceptives without barriers (e.g., ensuring that methods are available and accessible; provided in a quality setting and with full information; and offered equitably without discrimination) [2].

To date, there has been a lack of comprehensive, longitudinal data to adequately examine the role that improved supply-side programming has on actual increases in family planning use [5]. What little attention there has been has focused on quality and availability as supply-side interventions. Most of this evidence has relied on data from population-based surveys or not covered urban Africa [6–10], the target area for this study. Moreover, findings on the relationships between facility improvements and contraceptive uptake are somewhat contradictory.

For example, Bolam and colleagues (1998) found a significant increase in contraceptive uptake at 6 months post-partum associated with women exposed to facility-based post-partum FP health education, but their study was limited to one facility in Kathmandu, Nepal [6]. Using Demographic and Health Survey data for rural Egypt, Ali (2001) found that facilities with fewer providers trained in FP service provision and fewer available methods were associated with discontinuation of contraceptive pill use [7]. In Peru, Mensch, Arends-Kuenning and Jain (1996) linked Demographic and Health Survey data to a Situation Analysis Survey of facilities and found that quality of care, as measured using a scale that included method availability, provider training, unbiased providers, non-restrictive providers, information provided by providers, cleanliness, privacy, and availability of other reproductive health services, was positively associated with current contraceptive use (*p* = 0.053) [8]. On the other hand, Hennink and Clements (2005) used repeated cross-sectional surveys in the catchment areas of four new FP clinics in urban Pakistan and found that the new clinics influenced method choice among users of contraception, but did not have a significant impact on overall contraceptive prevalence [9]. Sherwood-Fabre and colleagues (2002) also used repeated cross-sectional surveys, and found similarly contradictory evidence about the success of a government project in six Russian cities designed to reduce abortion-related maternal mortality through physician training, informational/educational materials, and provision of contraceptive supplies, with contraceptive use increasing in one study city, but significantly decreasing in another [10]. Among the few studies using longitudinal data and conducted in an African context, Tumlinson et al. (2015) found that, in Kenya, some facility quality measures (providers asking about FP preferences and helping the client with method selection, as well as clients reporting that they were treated “very well”) were associated with current contraceptive use, but other measures (provider competence, follow-up, and integration of services) were not associated with current contraceptive use [11].

The purpose of this study is to examine the role that contraceptive availability and facility quality play in contraceptive uptake in urban areas in Nigeria, Kenya, and Senegal. We consider interventions undertaken by the Urban Reproductive Health Initiative (URHI), which sought to increase demand for FP, improve family planning service quality, and increase contraceptive availability in urban settings. Funded by the Bill & Melinda Gates Foundation (BMGF), the goal of the URHI was to significantly increase modern contraceptive use, particularly among the urban poor, in Uttar Pradesh, India, Nigeria, Kenya, and Senegal. The Measurement, Learning & Evaluation (MLE) project was the evaluation component of the URHI and was also funded by BMGF [12].

Other MLE studies demonstrate the impact of URHI demand generation and access to URHI supply-side programming on women’s reported contraceptive use in India and Nigeria [13, 14]. In this study, we focus on FP service quality and availability in the three African countries included in the MLE project. We use longitudinal facility data collected by MLE to consider whether the number of new FP acceptors, as well as the total number of FP users increased more at facilities with greater URHI program implementation between 2011 and 2014/2015.

## Methods

This study uses baseline and endline data collected by MLE during facility audits and individual provider interviews at facilities providing reproductive health and/or maternal and child health services in targeted cities in Nigeria, Kenya, and Senegal. Since the programs covered the entire cities with demand creation programming and interventions were undertaken in high-volume and targeted facilities, it was not possible to have a comparison group in this study. In all included facilities, facility audits recorded general facility characteristics, quality assurance procedures, and physical infrastructure and equipment. Provider interviews asked about FP training, knowledge of FP, and integration of FP with other services. At larger facilities, four providers offering relevant RH services were selected at random for provider interview. All providers were interviewed at facilities with four or fewer providers offering FP services.

In Senegal, baseline and endline surveys were conducted at all public and private facilities offering at least one reproductive health and/or maternal and child health service in the six program cities (Dakar, Guédiawaye, Pikine, Mbao, Mbour, and Kaolack), and included any new facilities at endline [15]. In Nigeria, surveys were conducted at all public facilities; all URHI program enrolled facilities; and at any private facility mentioned by women as a source of reproductive health and/or maternal and child health services at baseline in the six cities (Abuja, Benin City, Ibadan, Ilorin, Kaduna, and Zaria) [16]. In the three smaller Kenyan cities (Kisumu, Kakamega, and Machakos), the surveys were conducted at all facilities offering reproductive health services. In the two largest Kenyan cities (Nairobi and Mombasa), the surveys were conducted at all public facilities, all URHI program facilities, as well as private facilities identified by women as providers of reproductive health and/or other maternal and child health services [17]. In all countries, a goal was to identify and implement program activities in higher volume facilities; these included both public and private facilities. Activities in program facilities varied based on the level of facility and the needs of the facility for quality improvement. Baseline audits and interviews were conducted in 2011 in all three countries. Endline audits and interviews were conducted in 2014 in Kenya and Nigeria and in 2015 in Senegal.

We used facility audit questions to create dependent variables for this analysis. For the first dependent variable, we used the total reported number of new FP acceptors at a facility. For the second dependent variable, we used the total reported number of clients who received FP services at a facility. Kenya and Nigeria reported the number of new acceptors or total FP clients over the last 12 months. Senegal reported the number of new acceptors or total FP clients over the last 6 months, so we multiplied the number of clients by 2 to estimate the number of new acceptors or FP clients over the last 12 months. Since larger facilities are likely to have more FP clients, we controlled for facility size and a facility’s investment in family planning by dividing the number of new acceptors or total FP clients by the number of permanent reproductive health (RH) staff at a facility, as identified in the full staff roster in the facility audit. This created our two dependent variables: the number of new FP acceptors per RH provider in the last 12 months and the number of FP clients per RH provider in the last 12 months.

We used both facility audit and provider interview responses to identify URHI program elements in each country. In all three countries, the program had the same objectives related to the supply side: a) integrate family planning into maternal, newborn, child, and HIV services; and b) improve the quality of services in high volume facilities. Each country implemented these activities somewhat differently depending on their contex; however, all included some form of provider training, information and outreach to patients, and ensuring stock availability. Below, as we describe the measurement methods in each country, we provide greater depth on these distinctions. We ran individual fixed effects models for each program component and each dependent variable separately as descriptive statistics. The program typically implemented multiple improvements at a given facility simultaneously, which made it difficult to identify separate effects for each improvement. We therefore used principal components analysis to generate a score based on the country-specific program components. Principal component analysis can reduce the number of variables by creating a series of uncorrelated linear combinations of variables [18]. We used the first component scores [19], which allowed us to capture the variation in “dose” of activities among program facilities.

In Kenya, the program components score was created using seven program elements, drawing from the three main URHI implementation areas mentioned above. Provider training included: whole site FP training led by URHI (where all facility staff, regardless of role and position were trained on FP); and staff receiving URHI training on FP commodity management. Information and outreach included: having URHI supported community health workers at the facility; the number of informational/educational materials observed during the facility audit; and implementing URHI supported outreach programs. Ensuring method availability included: participation in contraceptive method redistribution (where FP methods were redistributed between facilities to ensure method availability); and URHI supported visits by clinical teams to provide long acting or permanent methods.

In Nigeria, the program components score was created using six program components. The provider training components included: any interviewed provider at a facility receiving in-service training from URHI at any time; any interviewed provider receiving a URHI monitoring visit (called Integrated Supportive Supervision – ISS); and any interviewed provider was a member of the URHI FP Provider Network (FPPN). Information and outreach components captured the number of FP informational/educational materials observed at the facility audit. Ensuring stock availability was measured based on whether there were any stock-outs of contraceptive methods in the last month, however, no specific intervention activity was undertaken in Nigeria to reduce stock-outs. Unique to Nigeria, URHI-sponsored facility renovation was also included.

In Senegal, the program components score was created using six program elements. The provider training components were the percentage of interviewed providers at a facility who received in-service FP training; and the percentage of interviewed providers who received training on the program-modified Systematic Screening of Client Needs tool that supported client referral. The information and outreach components included the facility undertaking URHI FP education programs at the facility; the number of informational/educational materials observed at the facility audit; and whether there was a URHI sponsored midwife at a facility. Ensuring stock availability included facility participation in the URHI Informed Push Model, a program unique to Senegal that seeks to improve the flow and availability of contraceptives..

Across all countries, greater than 88% of facilities interviewed at baseline were successfully interviewed at endline as well. Not all facilities provided answers to questions about program components and the number of new acceptors and FP users at both time points. For the number of new acceptors variable, missing data resulted in a loss of 39.9% of observations in Kenya, 46.2% of observations in Nigeria, and 52.0% of observations in Senegal. For the number of family planning users variable, missing data resulted in a loss of 41.5% of observations in Kenya, 42.2% of observations in Nigeria, and 50.7% of observations in Senegal. Most of this loss was due to low response to the questions about the number of new acceptors and FP users in all three countries at baseline; this may be a result of unavailability of records to answer the questions. Facilities for which full data were not available for both time periods were excluded from further analyses. Based on a logistic regression of which facilities were excluded, we found that facilities excluded from the model were significantly more likely to have been added at endline in all three countries; significantly more likely to be a private facility in Nigeria and Kenya; and were significantly likely to have a larger staff in Kenya. URHI-specific program components were set to 0 at baseline as the program did not yet exist. Table 1 presents the total number of facilities surveyed and the number included in these analyses.

**Table 1:** Description of program components at baseline (2011) and endline (2014/5) in full sample of facilities, MLE facility surveys from urban Kenya, Nigeria, and Senegal

	Baseline N	Baseline percentage	Endline N	Endline percentage
Kenya (Baseline *N* = 279; Endline *N* = 377)				
Participation in contraceptive method redistribution	0	0%	184	52.6%
Whole site FP training led by URHI	0	0%	161	43.3%
URHI supported community health workers	0	0%	125	33.6%
Any FP informational/educational materials observed	229	82.1	316	83.8%
URHI supported outreach programs	0	0%	110	29.4%
Any staff received URHI training on FP commodity management	0	0%	191	69.5%
URHI visits by clinical teams to provide long acting or permanent methods.	0	0%	179	48.0%
Nigeria (Baseline *N* = 400; Endline *N* = 385)				
Any provider received URHI training	0	0%	145	37.7%
Any provider received a URHI monitoring visit	0	0%	220	57.1%
Any provider was a member of the URHI FP Provider Network	0	0%	135	35.1%
URHI sponsored renovation	0	0%	217	65.8%
No stock-outs of contraceptive methods in the last month	311	77.8%	324	84.2%
Any FP informational/educational materials observed	272	68%	311	80.8%
Senegal (Baseline *N* = 205; Endline *N* = 249)				
Any providers who received in-service FP training	160	78.1%	201	80.7%
URHI sponsored midwife	0	0%	18	7.2%
URHI Informed Push Model	0	0%	146	63.5%
Facility-based URHI FP education programs	0	0%	123	53.5%
Any provider received training with the Systematic Identification of Client Needs tool.	0	0%	139	55.8%
Any FP informational/educational materials observed	141	68.8%	197	79.1%

We used fixed effects regression analysis to determine whether the total number of FP users or new acceptors per provider increased more at facilities with higher URHI program scores. Facility improvements were targeted to underserved areas, so standard regression methods might have resulted in a downward bias in the impact of the improvements. Fixed effects regression corrects for this source of bias and approximates the pre-test/post-test design in experimental setting by allowing each facility’s baseline score to serve as the reference point for its endline score, rather than using baseline scores from other better resourced facilities [20]. We used a panel sample of facilities for which data were available for independent and dependent variables for both baseline and endline. Facilities missing data from one or both time periods were not included in the matched panel [21]. We analyzed each country and each outcome separately, using Stata 14.1.

## Results

Descriptive statistics for program components and program outcomes are presented in Tables 1 and 2. Participation in URHI program components including availability of informational/education materials, experience of stock-outs, and provider training, changed during the study period in all three countries. Program outcomes also improved. The average number of new acceptors per reproductive health staff member increased over the four-year evaluation period from 99 to 168 in Kenya, from 21 to 51 in Nigeria, and from 51 to 65 in Senegal. The average overall number of FP users per reproductive health staff member increased from 308 to 417 in Kenya, from 53 to 102 in Nigeria, and more modestly in Senegal from 228 to 232.

**Table 2:** Description of program outcomes in panel facility sample, MLE facility survey in urban Kenya, Nigeria, and Senegal

	Baseline	Endline
Average number of new acceptors per reproductive health staff member in the last 12 months		
Kenya (197 facilities in panel sample)	99	168
Nigeria (210 facilities in panel sample)	21	51
Senegal (109 facilities in panel sample)	51	65
Average number of FP users per reproductive health staff member in the last 12 months		
Kenya (192 facilities in panel sample)	308	417
Nigeria (226 facilities in panel sample)	53	102
Senegal (112 facilities in panel sample)	228	232

Table 3 presents associations between individual URHI program components and program outcomes, as well as the number of facilities in the matched panel for each model. In separate fixed effects models, many program components were significantly associated with the number of new acceptors and the number of FP users per reproductive health staff member. Fewer individual program components were significantly associated with the number of new acceptors or FP users in Senegal than in the two other countries; this may be due to the high coverage of program activities across Senegal facilities and lack of variability in program implementation across the facility sample. We tried to estimate models with all interventions included as separate independent variables but the interventions were too highly correlated for us to be able to measure separate effects. This means that, in all three countries, because facilities often undertook multiple program interventions simultaneously, any association between implementation of one program component and an increase in new acceptors or FP users may be due to that program component, or to some combination of other program components implemented at the same time.

**Table 3:** Association between program individual components and the number of new acceptors and the number of FP users per reproductive health staff member, MLE facility survey

Intervention	Number of new acceptors in 12 months per staff member			Number of FP users in 12 months per staff member		
	Estimate	95%CI	*P*-value	Estimate	95% CI	*P*-value
Kenya: number of facilities in panel sample^a^	197			192		
*Intervention*						
Participation in contraceptive method redistribution	94	9–179	0.03	205	30–380	0.02
Whole site FP training led by URHI	68	12–124	0.02	132	18–247	0.02
URHI supported community health workers	71	13–129	0.02	169	50–287	< 0.01
Any FP informational/educational materials observed	19	−84 – 121	0.72	−55	−280 – 169	0.63
URHI supported outreach programs	64	6–122		116	−6 – 239	0.06
Any staff received URHI training on FP commodity management	84	33–135	< 0.01	118	16–222	0.02
URHI visits by clinical teams to provide long acting or permanent methods.	90	45–135	< 0.01	135	40–231	< 0.01
Nigeria: number of facilities in panel sample^a^	210			226		
*Intervention*						
Any provider received URHI training	59	30–89	< 0.01	83	31–135	< 0.01
Any provider received a URHI monitoring visit	40	15–65	< 0.01	86	43–128	< 0.01
Any provider was a member of the URHI FP Provider Network	51	20–81	< 0.01	120	68–171	< 0.01
URHI sponsored renovation	41	16–66	< 0.01	70	26–113	< 0.01
No stock-outs of contraceptive methods in the last month	37	4–70	0.03	67	10–124	0.02
Any FP informational/educational materials observed	52	7–97	0.02	84	10–157	0.03
Senegal: number of facilities in panel sample^a^	109			112		
*Intervention*						
Any providers who received in-service FP training	26	−7 – 59	0.12	73	−53 – 199	0.25
URHI sponsored midwife	46	6–85	0.02	63	−100 – 256	0.45
URHI Informed Push Model	20	5–35	< 0.01	30	−32 – 92	0.34
Facility-based URHI FP education programs	17	0–35	0.05	65	−4 – 135	0.07
Any provider received training with the Systematic Identification of Client Needs tool.	17	1–33	0.3	−6	−70 – 59	0.86
Any FP informational/educational materials observed	−1	−7 - 4	0.62	−7	−30 - 16	0.53

^a^Panel sample sizes vary by outcome due to missing data

We therefore used unweighted principal components analysis to create a dose score for each country. In all three countries, the mean dose score was 0.00, with a standard deviation of 1.67 in Kenya, 1.84 in Nigeria, and 1.64 in Senegal. Facilities with more program components have higher dose scores. The number of new acceptors increased significantly at facilities with higher program scores in all three countries. The mean number of new acceptors per provider in the last 12 months increased by 18 (95% CI: 7–29) in Kenya, 14 (95% CI: 8–20) in Nigeria, and 7 (95% CI: 3–12) in Senegal with a 1-point increase in program score. For the mean number of FP users per provider, significant increases were found only in Kenya and Nigeria. In particular, with a one-point increase in program score, the mean number of FP users per provider in the last 12 months increased by 31 (95% CI: 7–56) in Kenya and 26 (95% CI: 15–38) in Nigeria (Table 4).

**Table 4:** Associations between facility quality program components score and the number of FP users in the last year per reproductive health staff member at URHI program facilities, MLE facility survey

	Number of new acceptors per reproductive health staff member			Number of FP users per reproductive health staff member		
	Estimate	95% CI	*P*-value	Estimate	95% CI	*P*-value
Kenya	18	7–29	< 0.01	31	7–56	0.01
Nigeria	14	8–20	< 0.01	26	15–38	< 0.01
Senegal	7	3–12	< 0.01	12	−7-31	0.21

## Discussion

This analysis finds that URHI program interventions at reproductive health care facilities in urban Nigeria, Kenya, and Senegal have led to increased contraceptive uptake and use at those facilities. Facilities with a higher dose of program activities, as estimated by program component scores, had greater increases in both the number of new FP acceptors and the total number of FP users in urban settings in Nigeria and Kenya; increases were found for new FP acceptors in urban Senegal. These findings suggest that a combination of supply-side approaches will be needed to meet FP goals.

As programs seek to undertake family planning programming with a rights-based lens, it is worthwhile to consider the supply-side elements of quality and availability included in this paper. Notably, these elements do not capture all of the principles of a rights-based approach but they include those that were relevant to the URHI programs and were typically measured in 2010 when study materials were developed. Future studies need to develop measures of client agency and autonomy as well as discrimination towards clients to better inform how to address these in rights-based family planning programming as well as to assess their contribution to family planning adoption and continuation.

This study is not without limitations. One limitation of this study is that URHI tended to implement multiple intervention activities at a facility simultaneously, which meant that we were unable to measure the separate effects for each intervention (e.g., increasing number of methods available, training providers, or integration of services). Although we were unable to disentangle the role of individual interventions, separate models by program activity (Table 3) suggest that different program elements may be more successful in some contexts than in others. For example, informational/educational materials at facilities were associated with an increase in FP users in Nigeria, but not in Kenya or Senegal. On the other hand, provider training was significantly associated with higher numbers of FP users in Nigeria and Kenya, but not in Senegal.

In addition, while use of panel data better allows us to draw causal inference about URHI programming and facility-level uptake and use of FP methods, we are unable to rule out confounding by other time-varying factors that may be associated with both contraceptive uptake and service improvements. Further, a number of facilities were excluded from the analysis because of missing data at one or more time points. This increases the risk of selection bias, although the use of fixed effects models corrects somewhat for this bias. Most of the exclusions were due to missing data about the number of new FP acceptors or FP users at private facilities or at baseline. This limits our ability to draw inferences about facilities that were added to the study, and about private facilities. The response rate improved somewhat at endline, however, indicating that future surveys might reduce this problem by investing in interviewer training on how to ascertain the number of clients based on medical records and by quality control to ensure that these questions are answered fully despite the effort involved in answering them. Further, this analysis used the number of RH staff members to control for facility size in considering the number of new FP acceptors or users per facility. While the number of RH staff is an imperfect proxy for facility size, we believe that it better captures a facility’s investment in RH services, regardless of size. A final limitation of this study is that the Urban Reproductive Health Initiative also sought to increase demand for FP. While the demand side is considered in other studies, this analysis focused only on the supply environment, and may therefore underestimate the program’s effect on contraceptive uptake in the three countries.

## Conclusions

The findings from this study help fill a gap in the existing literature about the effectiveness of various supply-side interventions on contraceptive uptake and use in urban African settings, where many of the FP2020 focus countries are located. These findings can be used to inform future programs seeking to contribute to the ambitious goals set by FP2020 for these countries. While tailoring to the local environment, programs seeking to increase the number of new and continuing FP users should ensure that their programs include components to improve availability and quality of FP services including training providers, commodity security, integrated services, method availability, outreach activities, and information and education materials.

Care should be taken to guarantee that these components are executed well, and in a manner that helps clients exercise their right to make their own decisions about FP uptake and use. In addition, programs should consider strategies to address client agency and autonomy as well as discrimination towards clients. These may be key elements that could lead to greater increases in family planning method use and support attainment of FP2020 goals. Finally, improving supply of services should go hand-in-hand with creating a demand for FP services and building agency to use these services; this will ensure that improved services are used to meet users’ needs and desires. Undertaking multi-component supply- and demand-side programming as done by the URHI program should help to lead to increased FP use and support attainment of FP2020 goals.

## Abbreviations

BMGF: Bill and Melinda Gates Foundation
FP: Family planning
MLE: Measurement, learning, and evaluation program
RH: Reproductive health
URHI: Urban Reproductive Health Initiative
